# Evaluation of Stroke Knowledge Among High School Students: A Large Sample Cross‐Sectional Study From Kezhou, China

**DOI:** 10.1155/srat/4723338

**Published:** 2026-10-09

**Authors:** Qinyuan Zhu, Yanzhe Ji, Xuechun Guan, Tohetahan Zaire, Rui Lei, Wei Xiang, Hailan Qian

**Affiliations:** ^1^ School of Nursing and Rehabilitation Nantong University Nantong Jiangsu Province China ntu.edu.cn; ^2^ Funing County Hospital of Traditional Chinese Medicine Yancheng Jiangsu Province China; ^3^ School of Medicine Nantong University Nantong Jiangsu Province China ntu.edu.cn; ^4^ Nantong First People′s Hospital Nantong Jiangsu Province China nt2191.com; ^5^ The Affiliated Kezhou People′s Hospital of Nanjing Medical University Kezhou Xinjiang Uygur Autonomous Region China xjmu.edu.cn; ^6^ Department of Neurology Affiliated Hospital of Nantong University Nantong Jiangsu Province China ahnmc.com

**Keywords:** cross-sectional study, high school student, stroke, stroke awareness, stroke knowledge

## Abstract

**Objective:**

To evaluate stroke awareness among high school students in Kezhou—a multiethnic region with limited healthcare resources—and to provide evidence for targeted stroke prevention and treatment education.

**Methods:**

This cross‐sectional study utilized a self‐designed questionnaire to assess stroke‐related knowledge among high school students. Differences in stroke knowledge across demographic subgroups were analyzed, with a statistical significance set at *p* < 0.05.

**Result:**

A total of 2525 questionnaires were collected. Full awareness rates for stroke emergency maps, the “stroke 1‐2‐0” rapid identification method, stroke classification, and intravenous thrombolytic therapy for ischemic stroke were 1.90%, 9.98%, 16.79%, and 20.00%, respectively. The lowest awareness rates for stroke risk factors were smoking (10.65%), unhealthy diet (12.75%), and obesity (22.34%), whereas the least recognized warning symptoms included personality changes (11.92%), dizziness/gait instability (17.23%), and blurred/double vision (22.30%). Correct response rates for stroke visit time, thrombolysis time window, and emergency management were 72.95%, 41.11%, and 21.50%, respectively. Students with stroke‐affected relatives, urban residents, and those exposed to stroke education demonstrated significantly higher knowledge levels (*p* < 0.05). Kirgiz students showed poorer emergency response knowledge compared with other ethnic groups (*p* < 0.05). Preferred educational channels included social media (66.61%), bulletin boards (57.50%), and medical professionals′ lectures (54.77%).

**Conclusion:**

Kezhou high school students exhibited low stroke awareness, particularly regarding risk factors, the “stroke 1‐2‐0” identification method, emergency maps, and crisis management. It is recommended that stroke education be integrated into ethnic cultural activities, and that social media and posters on campuses and lectures by medical personnel be used to disseminate the “stroke 1‐2‐0” recognition method and improve regional stroke prevention and treatment capabilities.

## 1. Introduction

Stroke, including ischemic stroke and hemorrhagic stroke, is an acute cerebral circulatory disorder characterized mainly by neurological dysfunction [1]. The prevalence of stroke in China ranks highest in the world [2]. The standardized prevalence of stroke among Chinese people aged 40 years or older was 2.64% in 2021 [3]. Stroke is the leading cause of death and disability among adult residents in China [4]. The prevalence of stroke in China shows significant regional differences [5]. A study covering the incidence data of stroke in 32 provinces in China showed that there were high incidence zones of stroke in nine provinces in the north and west of China, including Xinjiang [6]. The overall cardiovascular and cerebrovascular disease burden in Xinjiang ranks second in China [7]. The unique culture, lifestyle, and dietary habits of Xinjiang Uygur Autonomous Region contribute to the high incidence of cardiovascular disease. Among people over 50 years old in Xinjiang Uygur Autonomous Region, the prevalence of stroke in Uygur is higher than that in Han [8]. Moreover, the prevalence of metabolic syndrome in this region has increased among both adults and adolescents, and the incidence of stroke is showing a trend of younger age [9].

Early identification and timely treatment after the onset of stroke are critical to reducing the mortality and disability rate of patients and the occurrence of complications. The shorter the time from the onset of ischemic stroke to reperfusion therapy, such as intravenous thrombolysis and endovascular treatment, the better the prognosis [10]. However, the thrombolysis rate and thrombectomy rate of stroke patients in China are less than 5%, and less than 20% of them arrive at the hospital within 3 h after stroke onset [11]. The main delay in emergency care for a stroke patient occurs between the onset of symptoms and the decision to seek medical care [12]. The time from stroke onset to emergency system activation is related to the public′s awareness of the disease [13]. Therefore, promoting the public to identify stroke and start the emergency system on time is particularly important to reduce the prehospital delay time and improve the outcome of stroke patients.

Stroke health education for middle school students is an effective means to disseminate scientific knowledge, enhancing students′ understanding of stroke and facilitating the transfer of this knowledge to their families and community [14, 15]. Kizilsu Kirgiz Autonomous Prefecture (Kezhou) is located southwest of Xinjiang. It is a multiethnic settlement with relatively backward economic development, short of medical resources, and relatively low health literacy among its residents. We hypothesize that high school students in Kezhou lack comprehensive knowledge of stroke. This study is aimed at evaluating stroke awareness among high school students in Kezhou, providing a foundation for developing targeted school‐based stroke health education programs.

## 2. Materials and Methods

### 2.1. Design and Participants

This study was a descriptive and cross‐sectional quantitative study. The study employed a cluster random sampling approach. Within the Kezhou administrative region are six senior high schools, each with approximately 800 first‐year students. All six schools were numbered as clusters, and three clusters were randomly selected using a random number table. Students who had resided in Kezhou for less than 3 years were excluded, whereas the remaining first‐year high school students were included in the survey. Data collection was conducted from August 23 to September 12, 2024, when centralized military training was carried out for first‐year students in high schools. Questionnaires were administered to students by investigators through face‐to‐face interviews. Prior to data collection, eight registered nurses with more than 3 years of hospital experience attended a unified training workshop covering standardized survey procedures, ethical considerations, face‐to‐face communication skills, on‐site real‐time quality checks, and student inquiry handling. First‐year high school students are of similar age and at the same educational stage, contributing to sample homogeneity. Permission for this survey was obtained from school administrators and local education authorities.

### 2.2. Methodology for Sample Size Calculation

The sample size for this study was determined using the OpenEpi 3.0 sample size calculation tool, a widely recognized and reliable online resource for estimating sample sizes in medical research [16]. According to the data on the official website of the People′s Government of Kizilsu Kirgiz Autonomous Prefecture, the total population of Kizilsu Kirgiz Autonomous Prefecture was 632,100 at the end of 2023. We set the confidence interval to 98%, the expected frequency percentage to 50%, and the effect to 1, and calculated the minimum sample size to be 2392. A total of 2525 participants were included in this study, of which 986, 608, and 931 were from the first, second, and third middle schools in Kezhou, respectively. Kezhou Prefecture is a multilingual region where Uyghur and Kyrgyz are widely spoken. However, standard Mandarin Chinese (Putonghua) is the sole language of instruction in all public high schools, and all first‐year students were fluent in Mandarin. The questionnaire was administered solely in Mandarin via face‐to‐face interviews; no translation was provided. Ethnicity was determined based on official government documentation (national ID card or household registration booklet), which record ethnicity under China′s 56‐category national classification system. Students reported the ethnicity as printed on their documents, and nurses verified this against the official record. This is the legally standardized method of ethnic classification in China, not based on phenotype or genetic assumption. In this sample, Uyghur and Kyrgyz participants were the two largest groups, reflecting the demographic composition of Kezhou Prefecture. The three largest groups (Han, Uyghur, and Kyrgyz) are reported individually; smaller groups were combined as “Other” to protect anonymity.

### 2.3. Ethics Approval

Written informed consent was obtained from all participants and/or their parents/guardians. All participants were voluntary and anonymous. This study was approved by the Institutional Review Board (No: 2023‐01‐05).

### 2.4. Measurements

The stroke knowledge questionnaire was developed by our research team based on a comprehensive review of multiple publicly available stroke knowledge assessment tools [17, 18]. The questionnaire was divided into three parts:

Part 1. General information questionnaire: This section consists of six questions covering age, gender, ethnicity, place of residence, prior exposure to stroke education services, and the presence of stroke patients in the family

Part 2. Stroke‐related knowledge and awareness questionnaire: This section includes 10 questions, seven of which are single‐choice questions assessing knowledge on stroke classification, the “stroke 1‐2‐0” rapid identification method, intravenous thrombolysis as a critical treatment for restoring cerebral blood flow in ischemic stroke patients, and awareness of the city′s stroke emergency map. Stroke symptom awareness was assessed using the “stroke 1‐2‐0 for China” mnemonic [19], a nationwide public health campaign developed by the China National Health Commission and the Chinese Stroke Association. The “1‐2‐0” stands for: “1”—look for one uneven face (facial asymmetry or drooping, particularly at the corner of the mouth); “2”—examine for arm weakness (ask the person to raise both arms parallel and check for unilateral weakness); and “0”—listen for absence of clear speech (slurred speech or difficulty expressing). The public is instructed to call 120 immediately if any of these symptoms are observed. This corresponds to China′s emergency number (120), enhancing public recall. Responses to these questions are categorized into four levels of understanding: “fully understand,” “heard of it but not sure,” “unaware but interested to learn,” and “unaware and not interested.” The remaining three questions pertain to optimal response times for seeking medical attention after symptom onset, the ideal window for administering intravenous thrombolysis in ischemic stroke cases, and appropriate emergency measures postonset. Participants had to select one correct answer from the options for these three questions. The questionnaire on stroke encompasses three multiple‐choice sections: synonyms for stroke, risk factors, and warning symptoms. The synonyms section provides five options: cerebrovascular event, cerebrovascular accident, cerebrovascular disease, cerebral hemorrhage, and cerebral infarction. The risk factors section includes nine options: hypertension, diabetes mellitus, hyperlipidemia, atrial fibrillation, obesity, physical inactivity, smoking, poor dietary habits, and excessive alcohol consumption. The warning symptoms section covers sudden facial drooping, weakness in the arms or legs, slurred speech, dizziness or difficulty walking, blurred or double vision, severe headache with vomiting, confusion, potential loss of consciousness or fainting, and changes in behavior. The questionnaire was evaluated by five stroke medical and nursing experts and one education expert, and its content validity was measured to be 0.933. Following pilot testing with 89 first‐year high school students from another school (not included in the main study), minor revisions were made to improve clarity and comprehension: Five items were reworded to simplify medical terminology, and two items had response options adjusted. These revisions were based on student feedback during piloting and were not related to the nurses. The Cronbach′s alpha of 0.703 was calculated on the final revised questionnaire using the main study data (*n* = 2525). The pilot sample was used to assess readability, not reliability; as the revisions did not alter the questionnaire structure, the prerevision and postrevision reliability are expected to be similar.

Part 3. Expected stroke awareness dissemination channels, including six options of teaching by medical staff, bulletin boards or stickers, paper media such as newspapers, periodicals, books, new media platforms like WeChat, Weibo, and TikTok, and teaching by school staff.

For each single‐choice question, the percentage of people who answered question correctly among the total sample size was calculated. For multiple‐choice questions, the response rate of each option (response rate = the number of people who chose a certain option / the total number of selected options for the question ×100*%*), the popularity rate (popularity rate = the number of people who chose a certain option / the number of people participating in the survey ×100*%*), and the proportion of items that answered correctly were calculated.

### 2.5. Data Collection Procedure

In this study, eight nurses who have worked in hospital for over 3 years were recruited to collect data and enter the school for face‐to‐face investigation after unified training. Before the formal investigation, 89 students in Grade 1 of a high school in Kezhou who did not participate in this study were selected for preinvestigation. Based on issues identified during the presurvey, the investigators received additional training, and the questionnaire was revised accordingly. Data collection was approved by the school′s academic affairs office and the respective class teachers. The researcher explained to the respondents that the survey was anonymous and would not be used commercially. The researcher also explained to the students the purpose and meaning of the survey and how to complete it. After the students filled in the questionnaire, the investigators checked whether the items were filled out completely, whether the handwriting was clear, and whether the whole article had logical errors. Questionnaires were checked for logical errors, defined as internally inconsistent, contradictory, or implausible responses (e.g., reporting no awareness of stroke yet correctly identifying multiple symptoms, or reporting an age outside the plausible range for high school students). Questionnaires with logical errors were excluded. Minor nonlogical errors, such as incomplete demographic fields or single skipped items, did not result in exclusion; missing data were handled via listwise deletion per analysis. After confirming that the questionnaire was filled out entirely and without logical errors, the investigators withdrew the questionnaire. In this study, 2650 questionnaires were distributed, and 2525 valid questionnaires were collected, with an overall recovery rate of 95.28%.

### 2.6. Statistical Analysis

The data of this study were input into the Epidata database by two project team members with medical backgrounds, and data entry training was done by two people using the back‐to‐back method. The inconsistent data entered by the two people were checked using the original data and then entered into the correct data. Data quality assurance involved two stages. First, the eight trained nurses performed a real‐time visual check of each completed questionnaire during face‐to‐face administration for missing responses, logical inconsistencies, and illegible entries; students were asked to clarify incomplete items on the spot. Second, two research assistants independently entered all data into EpiData 3.1, and discrepancies were resolved by reference to the original paper form. A third researcher performed a random audit of 10% of entries for accuracy. The data were imported into SPSS27.0 software, and the value range, abnormal value, and missing value of the data were checked and cleaned by researchers again. The Statistical Package for Social Sciences (SPSS) Version 27.0 software package was used to analyze the data. Mean ± standard deviation was used to describe the continuous variables such as age. Categorical variables such as gender, ethnicity, residence, and response to stroke knowledge were characterized by frequency and percentage. Pearson′s chi‐square test or Fisher′s exact test was used to compare the differences in knowledge of stroke among respondents with different characteristics. The significance level for all statistical tests was set at *p* < 0.05 (two‐tailed).

## 3. Results

### 3.1. Participant Characteristics

All 2525 participants were first‐year high school students, aged 15–19 (15.84 ± 0.59) years old. Among them, 1212 (48.00%) were male, 1573 (62.30%) were Uyghur, 1367 (54.14%) resided in urban areas, and 285 (11.29%) had a relative with stroke. Furthermore, 439 participants (17.39%) had been exposed to science popularization knowledge about stroke (see Table 1).

**Table 1:** Participants′ sociodemographic characteristics (*N* = 2525).

Variables	Group	Frequency	%
Gender	Male	1212	48.00
	Female	1313	52.00

Ethnic	Han	518	20.51
	Uyghur	1573	62.30
	Kirgiz	426	16.87
	Other	8	0.32

Residence area	Urban	1367	54.14
	Rural	1122	44.44
	Mountainous region	36	1.43

Relatives with stroke	Yes	285	11.29
	No	1845	73.07
	Unclear	395	15.64

History of stroke science knowledge learning	Yes	439	17.39
	No	1157	45.82
	Unclear	929	36.7

### 3.2. Participants′ Cognitive Level of Stroke Knowledge

Among the 2525 participants, there was a diverse range of knowledge regarding stroke. Specifically, 424 (16.79%) were aware that stroke is categorized into ischemic and hemorrhagic types; 252 (9.98%) knew the “stroke 1‐2‐0” rapid identification method; 505 (20.00%) understood intravenous thrombolysis therapy for ischemic stroke; and 48 (1.90%) were familiar with their city′s stroke emergency map. Furthermore, 1842 (72.95%), 1038 (41.11%), and 543 (21.50%) correctly identified the optimal time from symptom onset to treatment, the time window for intravenous thrombolysis in ischemic stroke, and the emergency management of stroke, respectively. (see Tables 2 and 3).

**Table 2:** Participants′ cognition of stroke‐related knowledge (*n*, %).

Variables (*N* = 2525)	Fully understand	Heard of it but not sure	Unaware but interested to learn	Unaware and not interested
Classification of stroke	424 (16.79)	41 (1.62)	2055 (81.39)	5 (0.2)
“Stroke 1‐2‐0” rapid identification method	252 (9.98)	859 (34.02)	1407 (55.72)	7 (0.28)
Intravenous thrombolysis is the first choice for ischemic stroke	505 (20.00)	1681 (66.57)	322 (12.75)	17 (0.67)
Stroke emergency map of the city	48 (1.90)	383 (15.17)	1945 (77.03)	149 (5.90)

**Table 3:** Participants′ accuracy in knowing stroke patients′ hospital arrival, thrombolysis time window, and emergency care (*n*, %).

Variables (*N* = 2525)	Options	Frequency	%
Optimal emergency time from stroke onset to clinic visit	The sooner, the better	1842	72.95
	Within 24 h	353	13.98
	No time limit	263	10.42
	Unknown	67	2.65

Optimal time for intravenous thrombolysis in ischemic stroke	Within 6 h	1038	41.11
	Within 24 hs	1103	43.68
	No time limit	127	5.03
	Unknown	257	10.18

Emergency management of stroke	Take a break and observe	1254	49.66
	Contacting family members	687	27.21
	Call 120 immediately	543	21.50
	Unknown	41	1.62

In the multiple‐choice question “synonyms of stroke,” the most prevalent option was cerebrovascular disease (43.05%); in the multiple‐choice question “risk factors for stroke,” hypertension had the highest prevalence rate (62.57%). In the multiple‐choice item “warning symptoms of stroke,” the highest prevalence rate was unilateral limb weakness or numbness (55.64%). Only eight (0.32%), three (0.12%), and three (0.12%) people knew all five concepts, nine risk factors, and six warning symptoms of stroke, respectively (see Tables 4 and 5).

**Table 4:** Participants′ response rate and prevalence of stroke synonyms, risk factors, and warning symptoms (*n*, %).

Variables		Response		Penetration rate (%) (*N* = 2525)
		Frequency	Rate (%)	
Synonyms of stroke	Cerebrovascular event	995	20.01	39.41
	Cerebrovascular accident	883	17.76	34.97
	Cerebrovascular disease	1087	21.86	43.05
	Cerebral hemorrhage	1052	21.16	41.66
	Cerebral infarction	955	19.21	37.82
	Summary	4972	100.00	196.91

Risk factors for stroke	Hypertension	1580	22.37	62.57
	Diabetes	877	12.42	34.73
	High blood lipids	1432	20.27	56.71
	Atrial fibrillation	700	9.91	27.72
	Obesity	564	7.99	22.34
	Lack of exercise	604	8.55	23.92
	Smoking	269	3.81	10.65
	Unhealthy diet	322	4.56	12.75
	Excessive drinking	715	10.12	28.32
	Summary	7063	100.00	279.71

Warning symptoms of stroke	Slanting of the mouth	1059	16.17	41.94
	Numbness or weakness in one limb	1405	21.46	55.64
	Not speaking clearly	1143	17.46	45.27
	Dizziness or unsteady walking	435	6.64	17.23
	Blurred or double vision in the eyes	563	8.60	22.29
	Severe headache, Vomiting	798	12.19	31.60
	Confusion or convulsions	844	12.89	33.43
	Change of disposition	301	4.60	11.92
	Summary	6548	100.00	182.38

**Table 5:** Proportion of the number of correct entries in stroke synonyms, risk factors, and warning symptoms by the participants.

Variables (*N* = 2525)	Number of correct entries	Frequency	%	Cumulative percentage (%)
Stroke synonym	One	956	37.86	37.86
	Two	871	34.50	72.36
	Three	526	20.83	93.19
	Four	164	6.50	99.68
	Five	8	0.32	100.00

Stroke risk factors	One	538	21.31	21.31
	Two	688	27.25	48.55
	Three	621	24.59	73.15
	Four	325	12.87	86.02
	Five	190	7.52	93.54
	Six	119	4.71	98.26
	Seven	33	1.31	99.56
	Eight	8	0.32	99.88
	Nine	3	0.12	100.00

Stroke risk factors	One	83	3.29	3.29
	Two	1238	49.03	52.32
	Three	878	34.77	87.09
	Four	278	11.01	98.10
	Five	45	1.78	99.88
	Six	3	0.12	100.00

### 3.3. Differences in Stroke Knowledge Among Participants With Different Characteristics

Bivariate analysis showed that there was no statistically significant difference in knowledge of stroke among participants of different genders (*p* > 0.05). There was a statistically significant difference in the percentage of correct responses on emergency stroke management among students of different ethnicities (*p* = 0.025); participants living in urban areas had higher rates of correct responses on preferred intravenous thrombolytic therapy for ischemic stroke (*p* < 0.001), stroke emergency maps in their city (*p* < 0.001), time to consultation (*p* = 0.036), and emergency stroke management (*p* = 0.008); students with friends and relatives with stroke had higher knowledge of the “stroke 1‐2‐0” rapid recognition method (*p* < 0.001), time to consultation (*p* = 0.010), and time window for intravenous thrombolysis (*p* < 0.001); students with a history of previous exposure to stroke science had higher knowledge of the “stroke 1‐2‐0” rapid recognition method (*p* < 0.001), time to consultation (*p* = 0.010), and time window for intravenous thrombolysis (*p* < 0.001). Students who have a previous history of exposure to stroke science popularization for the rapid identification method of “stroke 1‐2‐0” (*p* < 0.001), the preferred intravenous thrombolytic therapy for ischemic stroke (*p* < 0.001), the stroke emergency map of the city where it is located (*p* < 0.001), the visit time (*p* < 0.001), the time window of intravenous thrombolytic therapy (*p* < 0.001), and emergency response (*p* < 0.001). All have a relatively high correct response rate (Tables 6 and 7).

**Table 6:** Comparison of cognition of stroke type, rapid recognition method, intravenous thrombolysis, and stroke map among participants with different characteristics.

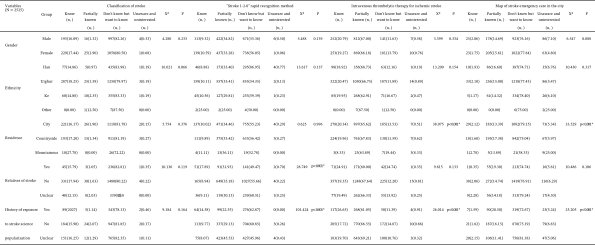

*Note:* The χ^2^ values of the significant comparisons are shown in italics.

^∗^ *p* < 0.001 indicates a statistically significant difference.

**Table 7:** Comparison of correct responses on time to stroke consultation, time to intravenous thrombolysis, and emergency management among participants with different characteristics.

Variables (*N* = 2525)	Group	From symptom onset to hospital time				Intravenous thrombolysis time window				Emergency management of stroke			
		Correct (*n*, %) (know)	Incorrect (*n*, %) (Unknown)	*X* ^2^	*p*	Correct (*n*, %) (know)	Incorrect (*n*, %) (Unknown)	*X* ^2^	*p*	Correct (*n*, %) (know)	Incorrect (*n*, %) (Unknown)	*X* ^2^	*p*
Gender	Male	333 (27.48)	879 (72.252	0.214	0.644	502 (41.42)	710 (58.58)	0.093	0.761	248 (20.46)	964 (79.54)	1.502	0.220
	Female	350 (26.66)	963 (73.34)			536 (40.82)	777 (59.18)			295 (22.47)	1018 (77.53)		

Ethnicity	Han	375 (72.39)	143 (27.61)	4.667	0.198	319 (61.58)	199 (38.42)	7.528	0.057	105 (20.27)	413 (79.73)	**9.331**	**0.025** ^∗^
	Uighur	1167 (74.19)	406 (25.81)			894 (56.83)	679 (43.17)			363 (23.08)	1210 (76.92)		
	Ke	294 (69.01)	132 (30.99)			269 (63.15)	157 (36.85)			72 (16.90)	354 (83.10)		
	Other	6 (75.00)	2 (25.00)			5 (62.50)	3 (37.50)			3 (37.50)	5 (62.50)		

Residence	City	1024 (74.91)	343 (25.09)	**6.649**	**0.036** ^∗^	558 (40.82)	809 (59.18)	4.257	0.119	325 (23.77)	1042 (76.23)	**9.633**	**0.008** ^∗^
	Countryside	795 (70.86)	327 (29.14)			471 (41.98)	651 (58.02)			213 (18.98)	909 (81.02)		
	Mountainous	23 (63.89)	13 (36.11)			9 (25.00)	27 (75.00)			5 (13.89)	31 (86.11)		

Relatives of stroke	Yes	218 (76.49)	67 (23.51)	**9.158**	**0.010** ^∗^	133 (46.67)	152 (53.33)	**31.654**	**p** < 0.001^∗^	74 (25.96)	211 (74.04)	3.816	0.148
	No	1359 (73.66)	486 (26.34)			792 (42.93)	1053 (57.07)			385 (20.87)	1460 (79.13)		
	Unclear	265 (67.09)	130 (32.91)			113 (28.61)	282 (71.39)			84 (21.27)	311 (78.73)		

History of exposure to	Yes	319 (72.67)	120 (27.33)	**65.721**	**p** < 0.001^∗^	365 (31.55)	792 (68.45)	**87.960**	**p** < 0.001^∗^	276 (23.85)	881 (76.15)	**18.737**	**p** < 0.001^∗^

Stroke science	No	763 (65.95)	394 (34.05)			193 (43.96)	246 (56.04)			110 (25.06)	329 (74.94)		

Popularization	Unclear	760 (81.81)	169 (18.19)			480 (51.67)	449 (48.33)			157 (16.90)	772 (83.10)		

^∗^ *p* < 0.05 indicates a statistically significant difference.

### 3.4. Participants′ Expectation of Popularizing Stroke Knowledge

The expected ways of stroke science popularization were new media (66.61%), bulletin boards (57.50%), teaching by medical staff (54.77%), teaching by school staff (49.31%), paper media (45.90%), and television and radio (32.55%) (Table 8).

**Table 8:** Participants′ expectation of popularizing stroke knowledge.

Variables		Response		Penetration rate % (*N* = 2525)
		Frequency	Rate (%)	
The desired way of popular science	Lectures by medical staff	1383	17.86	54.77
	Bulletin boards, posters	1452	18.75	57.50
	Paper media	1159	14.97	45.90
	Social media	1682	21.72	66.61
	Television and radio	822	10.62	32.55
	Lectures by school staff	1245	16.08	49.31
	Summary	7743	100.00	306.64

## 4. Discussion

As a global public health problem, stroke is one of the leading causes of adult disability and death. Improving public awareness of stroke is an important part of reducing the incidence and improving the prognosis of stroke. This study assessed stroke awareness among high school students in Kezhou.

This investigation indicated that the knowledge level of stroke among high school students in Kezhou is low. The full awareness rate of the “1‐2‐0” rapid identification method for stroke is only 9.98%, and the full awareness rate of the stroke emergency map in the city where it is located is as low as 1.90%. The complete awareness rates for the types of stroke and the preferred intravenous thrombolytic therapy for ischemic stroke were 16.79% and 20.00%, respectively. Among the student population, only 21.50% of the students are aware that they need to call 120 immediately upon discovering a stroke patient. In terms of risk factors for stroke, the awareness rates of students regarding smoking, unhealthy diet and obesity were 10.65%, 12.75%, and 22.34%, respectively. The awareness rates of stroke warning symptoms such as personality changes, dizziness or unsteady walking, blurred vision, or double vision were only 11.92%, 17.23%, and 22.30%, respectively. Such a low level of stroke awareness is mainly related to the relatively backward economic conditions and shortage of medical resources in Kezhou, which is located in the westernmost region of China. A study reported that the level of stroke management among primary health care providers in Kezhou was mild to moderate, and the awareness rate of stroke management time window was only 39.7% [20]. Similarly, a stroke health education program among primary school students from an ethnic minority area in Yunnan, China, showed that, before the intervention, 215 students had 0% accuracy in identifying the definition of stroke, its sequelae, and “stroke 1‐2‐0” quickly [21]. Furthermore, a survey also pointed out that rural residents in northwest China have a relatively low accuracy rate in their stroke awareness [18]. As a control, the awareness rates of stroke knowledge, risk factors, and early symptoms among Italian high school students were 76.5%, 62.4%, and 65.9%, respectively [22]. Besides, geographical location is also an important factor that causes the overall low stroke awareness among high school students. Kezhou is located in an alpine desert area, which is sparsely populated, and the distribution of medical and educational resources is very uneven. Notably, we found that Kirgiz students′ awareness of emergency treatment of stroke was significantly lower than that of other nationalities (*p* < 0.05). This difference is mainly related to the fact that the Kyrgyz people mostly live in remote mountainous areas, and their overall health literacy level is much lower than the average level of Chinese residents. As an example, a recent survey indicated that the smoking and drinking rates among the Kirgiz people remain high. Among the underage group, the youth group, the middle‐aged group, and the elderly group, the proportions of people who smoke more than five cigarettes a day for more than 6 months are 29.60%, 32.90%, 37.26%, and 36.27%, respectively [23]. Demonstrations of stroke risk‐related behaviors such as parents′ smoking have weakened students′ sensitivity to stroke risk factors and early warning symptoms.

This survey also highlighted the necessity and possibility of strengthening the popular science education of stroke‐related knowledge in high school students. This study found that relatives of stroke, living in the city, and exposure to popular science positively affected students′ stroke knowledge level (*p* < 0.05). Students who have family members with stroke patients may have more opportunities to be exposed to stroke‐related information, such as learning about stroke knowledge in family discussions. In addition, urban residents′ health serviceability is better than rural and mountainous areas, so urban students could have more opportunities to access information about stroke and emergency treatment. Meanwhile, popular science could effectively improve the stroke knowledge of students, and students with a history of exposure to popular science had a higher stroke knowledge level than those without (*p* < 0.001).

The unhealthy lifestyle model of family members and the lack of health literacy of elders significantly limit the efficiency of health knowledge transmission between generations, and high school students are an important link to rebuilding health knowledge transmission, especially for underdeveloped areas such as Kezhou. This study suggested that public health authorities should carry out targeted popular science activities on the risk factors and warning symptoms of stroke in high school students′ families, especially in promoting the “stroke 1‐2‐0” rapid identification method, the stroke emergency map of the city, and intravenous thrombolysis. Popular science workers can try to adapt stroke prevention knowledge into minority fables, proverbs, and rap art and spread it in schools, families, and communities. These measures will promote the public′s awareness of stroke risk factors and early warning symptoms, thereby reducing the incidence of stroke and assisting the public in identifying strokes on time and seeking medical treatment promptly to improve therapeutic effects and prognosis.

The results of this study showed that the popular science methods that students expected were social media, bulletin boards, and teaching by medical staff. With the rapid development of new media such as WeChat, Xiaohongshu, TikTok, and Weibo, social media platforms have become indispensable to People′s Daily life. Thus, health management institutions should fully consider the characteristics of high school students and minority cultures, expand the channels of stroke science popularization, innovate the methods of popular science education, and build a new media matrix of stroke prevention and treatment that integrates the characteristics of national culture (including short videos, live broadcasts, and AI interaction). Secondly, we should conduct community outreach publicity and distribute promotional materials on the “stroke 1‐2‐0” rapid identification method, stroke risk factors, low‐salt and low‐fat diet, quitting smoking, and limiting alcohol consumption and weight control in schools, bazaars, and ethnic festival celebration venues. Third, the local government cooperates with public health management institutions, education departments, and medical experts to carry out popular science education on stroke in schools to improve stroke awareness and encourage students′ enthusiasm for spreading stroke knowledge.

Although this study only analyzed the level of stroke awareness among high school students in Kezhou and proposed improvement measures accordingly, we believe the data of this study and the proposed measures could have reference value not only for the northwest region of China but also for regions with similar natural conditions and economic and cultural characteristics.

## 5. Limitations

This study′s sample was limited to first‐year high school students, which may not fully represent the target population. Additionally, reliance on self‐reported data might overestimate participants′ actual knowledge levels. As a cross‐sectional study, it cannot establish causal relationships. Future research should expand the scope of participants and implement targeted interventions based on these findings to validate the conclusions further.

## 6. Conclusion

This study describes the low level of stroke knowledge among first‐year high school students in Kezhou, Xinjiang. There is much room for improving the participants′ knowledge of stroke first‐aid maps and the “stroke 1‐2‐0” rapid recognition method, stroke types, intravenous thrombolytic therapy, emergency response, and blind spots in the knowledge of stroke risk factors and early warning symptoms. It is recommended that governmental departments, in conjunction with public health management and education departments, take into account the cultural specificities of high school students and ethnic minorities and carry out stroke health education, stroke risk factor screening, and standardized intervention programs based on students′ families, to enhance the awareness and ability of stroke prevention and treatment among people in Kezhou and even the whole of Xinjiang and to reduce the incidence of stroke.

## Author Contributions

Qinyuan Zhu, Yanzhe Ji, and Hailan Qian have contributed to the work equally and should be regarded as cofirst authors.

## Funding

This study was supported by the Open Project of the State Key Laboratory for Co‐Construction of Causes and Prevention of High Morbidity in Central Asia (SKL‐HIDCA‐2024‐KZ4) and the Science and Technology Bureau of Nantong City (JC2021096).

## Conflicts of Interest

The authors declare no conflicts of interest.

## Data Availability

The data that support the findings of this study are available from the corresponding author upon reasonable request.
